# Secondhand Smoke in the Workplace Is Associated With Depression in Korean Workers

**DOI:** 10.3389/fpubh.2022.802083

**Published:** 2022-04-26

**Authors:** Seunghan Kim, Juyeon Oh, Byungyoon Yun, Ara Cho, Juho Sim, Jin-Ha Yoon

**Affiliations:** ^1^Department of Preventive Medicine, Yonsei University College of Medicine, Seoul, South Korea; ^2^Department of Public Health, Graduate School, Yonsei University, Seoul, South Korea; ^3^Department of Occupational Health, Graduate School of Public Health, Yonsei University, Seoul, South Korea; ^4^The Institute for Occupational Health, Yonsei University College of Medicine, Seoul, South Korea

**Keywords:** secondhand smoking, depression, tobacco smoke pollution, working environment, KNHANES

## Abstract

**Background:**

Smoking negatively affects health, and previous studies argue that secondhand smoke (SHS) has a significantly negative health effect. We investigated whether SHS in the workplace influences workers' depression.

**Methods:**

Three years of data (2014, 2016, and 2018) from the Korean National Health and Nutrition Examination Survey were analyzed. Participants who were not current smokers were classified into the occupational SHS exposed and non-exposed groups. Multivariate logistic regression was performed to estimate odds ratios (ORs) and 95% confidence intervals (CIs) by adjusting various covariates. Stratified analysis with variables, such as year, sex, occupational classification, average working hours, was additionally performed.

**Results:**

The crude ORs of depression was 1.51 (95% CI: 1.27–1.80), and the fully adjusted OR with all the covariates was 1.57 (95% CI: 1.30–1.88). This indicated a significant relationship between occupational SHS and depression. The ORs increased every 2 years: 1.07 (95% CI: 0.79–1.44) in 2014, 1.88 (95% CI: 1.34–2.64) in 2016, and 2.07 (95% CI: 1.43–2.99) in year 2018. Stratification analysis also showed a significant association between SHS and depression among those in the prolonged work hours group and male employees, as well as blue- and white-collar workers.

**Conclusion:**

SHS in the workplace was significantly associated with workers' depression. Our study provides insights into the impact of exposure to SHS for workers and provides a basis for further research and policy-making in this field.

## Introduction

Smoking has various harmful effects on health and is known to cause cardiovascular diseases, such as ischemic heart disease, pulmonary diseases, including chronic obstructive pulmonary disease (COPD), and different cancers (1–4). According to the Global Burden of Disease, smoking accounted for 8.71 million deaths every year between 2010 and 2019 (5). Furthermore, secondhand smoke (SHS), caused by side-stream smoke (emitted from burning tobacco) and mainstream smoke (exhaled by the person who smokes), is an important health issue as it has been proven that people who inhale secondhand smoke are exposed to more than 60 carcinogens, including nicotine, N-nitrosamines, and aldehydes (6, 7). Side-stream smoke is largely responsible for the negative effect of SHS on health (8).

Previous research shows a significant relationship between SHS exposure and mental health outcomes (9). Some researchers have postulated possible mechanisms to explain the relationship between SHS and depression. One of the potential mechanisms is the induction of oxidative stress and systemic pro-inflammatory responses caused by tobacco smoke, which results in systemic inflammation and the circulation of inflammatory mediators. The mediators may interact with, and activate, cytokine receptors in brain endothelial cells, resulting in autoantibodies against cell junction and neural proteins. Autoantibodies cause neuroinflammation, oxidative stress in the brain, and neurochemical alterations, these inflammatory variables have been linked to the development of depressive disorders (10, 11). Another potential mechanism is the mesolimbic reward circuit with dopamine. After being exposed to SHS, nicotine absorbed into the body stimulates the brain reward circuit resulting in dopamine release. The exposure to unwanted SHS has a significant effect on the NAc and ultimately leads to instability of the dopaminergic system. The nicotine contained in tobacco smoke, which can directly affect SHS, causes instability of the dopamine system, resulting in psychological stress and depression (12).

Exposure to SHS in the workplace is a serious public health concern. Given that people spend most of their day at a workplace, SHS might be a noteworthy factor for workers' health (13, 14). Therefore, several measures have been taken to prohibit smoking in the workplace and public places. Several international conventions and guidelines have been adopted under the auspices of the International Labor Organization that emphasize workers' rights to a safe and healthy working environment. The convention is legally required to respect all workers' rights to breathe clean air and to regulate measures for the prevention, control, and protection against occupational risks in the workplace caused by air pollution (15). In the United States, many states have enacted smoke-free laws in the workplace and public spaces for people who never smoked (16). In Korea, there is a higher prevalence of SHS exposure than many other developed countries (17). To prevent this, non-smoking areas and smoking areas must be distinguished indoors, and in smoking areas, facility standards such as ventilation facilities must be satisfied. In addition, education on the harmful effects of active smoking and secondhand smoking should be provided (18, 19).

However, despite these efforts, SHS in the workplace remains a significant problem. According to previous studies, SHS is significantly associated with workers' health problems, including lung cancer and mental health (20–23). In particular, mental health issues, such as depression, can lead to industrial accidents, absenteeism, and low productivity (24). Moreover, mental health problems of workers can lead to significant industrial-economic burdens. In the United States, the percentage of economic costs because of depression among workers increased from 48 to 61% between 2010 and 2018 (25). According to a South Korean study, the estimated economic loss per month for workers with depression in 2008 was $135 (26). However, there are insufficient studies regarding the association between SHS in the workplace and depression in Korean workers (22, 27, 28).

Hence, this study aimed to investigate the relationship between SHS and depression in paid workers with job-related covariates, using data from the Korea National Health and Nutrition Examination Survey (KNHANES).

## Materials and Methods

### Study Population

The KNHANES is a nationwide cross-sectional survey conducted on National Health Promotion (18). The purpose of this survey is to establish nationally representative and reliable statistics on health, to evaluate the National Health Promotion comprehensive plan, and to develop health promotion programs.

The most recent data from the Population and Housing Census available at the time of designing the sample was used as the basic extraction frame of KNHANES and public housing price data was added to supplement the frame and improve the population inclusion rate. As for the sampling, a two-step stratified colony sampling method was used with survey district and household as the primary and secondary sampling units. The KNHANES also collects survey data through household member confirmation surveys, health surveys, screening surveys, and nutrition surveys. The household member confirmation surveys are basic surveys to identify the status of all residences and households in the area selected through sample design and select households to participate in health surveys, examinations and nutrition surveys.

**Figure 1:**
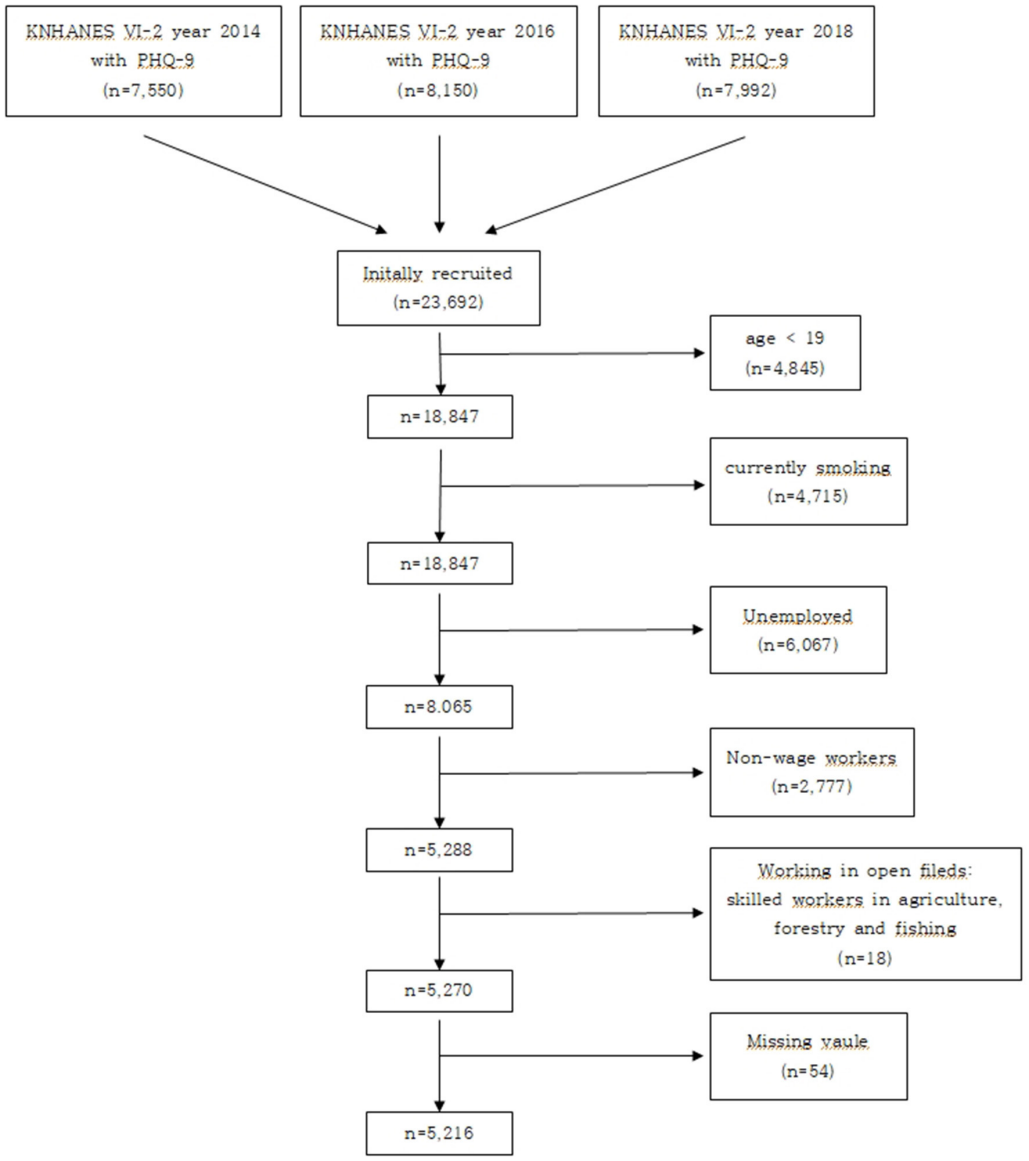
The schematic flow of the inclusion and exclusion criteria of participants.

For this study, KNHANES VI-VII (2013–2018) survey data were used. The survey used population and housing census data as the extraction mold to confirm systematic sampling. Excluding data without the Patient Health Questionnaire 9-item depression module (PHQ-9), data from the KNHANES VI-2 (2014), KNHANES VII-1 (2016), and KNHANES VII-3 (2018) were used. The initially recruited participants comprised 23,692 individuals, with 7,550 in 2014, 8,150 in 2016, and 7,992 in 2018. The exclusion criteria were as follows: participants <19 years old (*n* = 4,845), currently smoking (*n* = 4,715), unemployed (*n* = 6,067), non-wage workers (*n* = 2,777), working in open fields (skilled workers in agriculture, forestry, and fishing, *n* = 18), and those with missing variables or values (*n* = 54). Ultimately, 5,216 participants were included in the study. The detailed schematic flow of the participant selection process is summarized in Figure 1.

The study was conducted according to the guidelines of the Declaration of Helsinki (1975) and approved by the Institutional Review Board of Severance Hospital (Severance IRB: 2021-2508-001). The KNHANES VI is administered by the Korea Center for Disease Control and Prevention (KCDC) and approved by the KCDC Institutional Review Board (2013-12EXP-03-5C and 2018-01-03-P-A). Each participant voluntarily participated and provided a signed written consent before participating in the study.

### Definitions of Covariates

Among wage workers who do not currently smoke, those who answered “yes” to the question “Are you exposed to secondhand smoke at work?” were defined as those exposed to occupational SHS. Occupational SHS was used as an independent variable. This study used the PHQ-9 to assess depression, which is the outcome of this study. The PHQ-9 has been widely used and validated as a screening tool for depression (29). Participants with a total score of ≥5 in the PHQ-9 were considered to belong to the group with depression.

Continuous variables such as age, body mass index (BMI), income, average working hours per week and categorical variables such as sex, current education level, marital status, occupation, strength exercise, shift work, monthly drinking rate, and high-risk drinking, were used as covariates for adjustment. BMI was classified into four groups: “underweight (<18.5),” “normal (18.5–22.9),” “overweight (23–24.9),” and “obese (≥25)” (30). Income quartiles were divided into four groups: “low”, “low intermediate”, “high intermediate”, and “high”. Average working hours per week were classified into “short” and “long” based on whether participants worked below or above 52 h. Sex was defined as “male” and “female”. Marital status was defined as “single” and “married”. Current education level was divided into four groups: “elementary school”, “junior high school”, “high school” and “college graduate”. Participants working during the day were classified as “does not engage in shift work” and the others in “does shift work.” Occupation was classified into three groups. Managers, experts, related workers, and office workers as “white-collar,” service and sales personnel as “pink-collar,” and, finally, technicians, machine operators, assembly workers, and simple labor workers as “blue-collar.” Participants who engaged in strength exercise more than once a week were classified as a “strength exercise” group. The monthly drinking rate was divided into two groups, “yes” and “no”, depending on whether participants drank at least once a month over the past year. High-risk alcohol consumption was defined as an average consumption of seven drinks or more for men and five drinks or more for women per time and drinking at least twice a week.

### Statistical Analyses

Baseline characteristics of participants were examined by the *t*-test for continuous variables and chi-squared test for categorical variables. Crude odds ratios (ORs) with 95% confidence intervals (CIs) and adjusted ORs with 95% CIs were calculated using logistic regression. All analyses were two-sided, and a *p* < 0.05 was considered significant. For whole statistics, R program version 4.0.5 was used as software.

## Results

Participants' characteristics at the baseline by occupational SHS exposure and non-exposure are displayed in Table 1. Workers who were exposed to SHS were mostly men from the low and upper-middle income groups who worked longer hours. They formed the majority of blue- and white –collar workers with high school or college education. Those who were not exposed were mostly white-collar workers with college education who appeared to consume more alcohol than those who were exposed to SHS. The mean age and standard deviation (SD) were 44.4 years (SD = 14.1) in the exposed group and 46.3 years (SD = 14.7) in the non-exposed group (*p* < 0.001). There were 52.4 and 34.4% men in the exposed and non-exposed groups, respectively. The percentage of first and third quartiles of income (low, upper intermediate income) were significantly greater in the exposed group (24.1, 28.2%) than in the non-exposed group (17.6, 27.1%, *p* < 0.001). Similarly, participants who were overweight and obese, were greater in the exposed group (23.3, 33.4%) than in the non-exposed group (23.3, 29.8%, *p* = 0.042). The mean and SD of average working hours per week was 41.9 h (SD = 15.2) for the exposed group and 36.8 h (SD = 15.7) for the non-exposed group. Most participants in the exposed (71.6, 86.3%) and non-exposed groups (75.5, 92.4%) did not engage in strength exercise once a week or have high-risk alcohol consumption, however, some participants (65.1, 54.5%) in the exposed and non-exposed groups consumed alcohol on a monthly basis. Among the participants in the exposed and non-exposed groups, 76.3 and 79.9% were married, 35.6 and 43.8% of those in the exposed group and 30.9 and 48.8% of those in the non-exposed group were high school and college graduates, respectively. A significantly higher proportion of participants were white- and blue-collar workers in the exposed and non-exposed groups (respectively, 42.1% and 38.4% and 51.0% and 30.6%), a higher percentage of participants did not engage in shift work in both the exposed and non-exposed groups (82.1 and 83.8%, respectively).

**Table 1:** Characteristics of participants at the baseline.

	**Non-exposed**	**Exposed**	***p*-value**
Age			<0.001
Mean (SD)	46.3 (14.7)	44.4 (14.1)	
Sex			<0.001
Male	1,449 (34.4%)	524 (52.4%)	
Female	2,767 (65.6%)	476 (47.6%)	
Income quartiles			<0.001
Low income	742 (17.6%)	241 (24.1%)	
Low intermediate	1,059 (25.1%)	241 (24.1%)	
Upper intermediate	1,142 (27.1%)	282 (28.2%)	
Advanced	1,273 (30.2%)	236 (23.6%)	
Education			0.004
Elementary school	515 (12.2%)	110 (11.0%)	
Junior high school	342 (8.1%)	96 (9.6%)	
High school	1,302 (30.9%)	356 (35.6%)	
College graduate	2,057 (48.8%)	438 (43.8%)	
Marital status			0.015
Single	846 (20.1%)	237 (23.7%)	
Married	3,370 (79.9%)	763 (76.3%)	
Average working hours per week			<0.001
Mean (SD)	36.8 (15.7)	41.9 (15.2)	
Does shift work			0.21
No	3,533 (83.8%)	821 (82.1%)	
Yes	683 (16.2%)	179 (17.9%)	
BMI			0.042
Underweight	182 (4.3%)	31 (3.1%)	
Normal	1,810 (42.9%)	402 (40.2%)	
Overweight	968 (23.3%)	233 (23.3%)	
Obese	1,256 (29.8%)	334 (33.4%)	
Occupation			<0.001
White-collar	2,151 (51.0%)	421 (42.1%)	
Pink-collar	774 (18.4%)	195 (19.5%)	
Blue-collar	1,256 (30.6%)	384 (38.4%)	
Strength exercise			0.009
No	3,185 (75.5%)	716 (71.6%)	
Yes	1,031 (24.5%)	284 (28.4%)	
Monthly drinking			<0.001
No	1,917 (45.5%)	349 (34.9%)	
Yes	2,299 (54.5%)	651 (65.1%)	
High-risk alcohol consumption			<0.001
No	3,896 (92.4%)	863 (86.3%)	
Yes	320 (7.6%)	137 (13.7%)	

**Figure 2:**
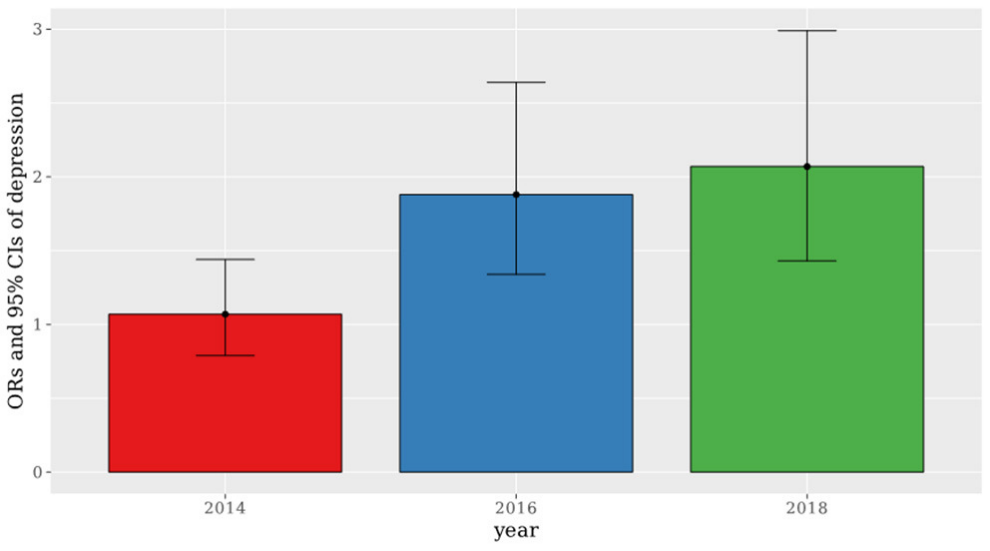
Time series ORs and 95% CIs for depression by secondhand smoke.

Table 2 presents the crude and adjusted ORs of depression obtained by logistic regression. The crude OR was 1.51 (95% CI: 1.27–1.80). Even after completely adjusting all the covariates, including sex, age, BMI, strength exercise, monthly drinking, high-risk alcohol consumption, income quartiles, education, marital status, average working hours per week, shift work, and occupation, the adjusted OR was 1.57 (95% CI: 1.30–1.88). Figure 2 shows the completely adjusted ORs of depression in a period sequence, stratified into 2014, 2016, and 2018. The ORs (95% CIs) were 1.07 (0.79–1.44) in 2014, 1.88 (1.34–2.64) in 2016, and 2.07 (1.43–2.99) in 2018.

**Table 2:** Odds ratios of occupational secondhand smoke and depression (adjusted and not adjusted).

	**Crude**		**Adjusted**	
Occupational passive smoke	1.51 (1.27–1.80)	1.70 (1.42–2.04)	1.68 (1.40–2.01)	1.57 (1.30–1.88)
Sex				
Male		1.00 (reference)	1.00 (reference)	1.00 (reference)
Female		2.10 (1.77–2.49)	2.15 (1.79–2.59)	2.16 (1.78–2.63)
Age				
Young		1.00 (reference)	1.00 (reference)	1.00 (reference)
Old		0.65 (0.56–0.76)	0.69 (0.59–0.80)	0.81 (0.65–1.00)
BMI				
Underweight			1.00 (reference)	1.00 (reference)
Normal			0.66 (0.47–0.92)	0.64 (0.45–0.89)
Overweight			0.59 (0.41–0.84)	0.56 (0.39–0.81)
Obese			0.72 (0.51–1.03)	0.67 (0.47–0.95)
Strength exercise				
No			1.00 (reference)	1.00 (reference)
Yes			0.91 (0.75–1.09)	0.92 (0.76–1.11)
Monthly drinking				
No			1.00 (reference)	1.00 (reference)
Yes			1.06 (0.90–1.25)	1.10 (0.93–1.30)
High-risk alcohol consumption				
No			1.00 (reference)	1.00 (reference)
Yes			1.50 (1.15–1.95)	1.49 (1.14–1.94)
Income quartiles				
Low income				1.00 (reference)
Low intermediate				0.80 (0.65–0.99)
Upper intermediate				0.61 (0.49–0.76)
Advanced				0.47 (0.38–0.60)
Education				
Elementary school				1.00 (reference)
Junior high school				0.69 (0.49–0.97)
High school				0.64 (0.49–0.84)
College graduate				0.73 (0.54–0.99)
Marital status				
Single				1.00 (reference)
Married				0.61 (0.49–0.76)
Average working hours per week				
Short				1.00 (reference)
Long				1.32 (1.06–1.64)
Does shift work				
No				1.00 (reference)
Yes				1.20 (0.98–1.46)
Occupation				
White-collar				1.00 (reference)
Pink-collar				1.06 (0.84–1.34)
Blue-collar				1.05 (0.83–1.34)

**Figure 3:**
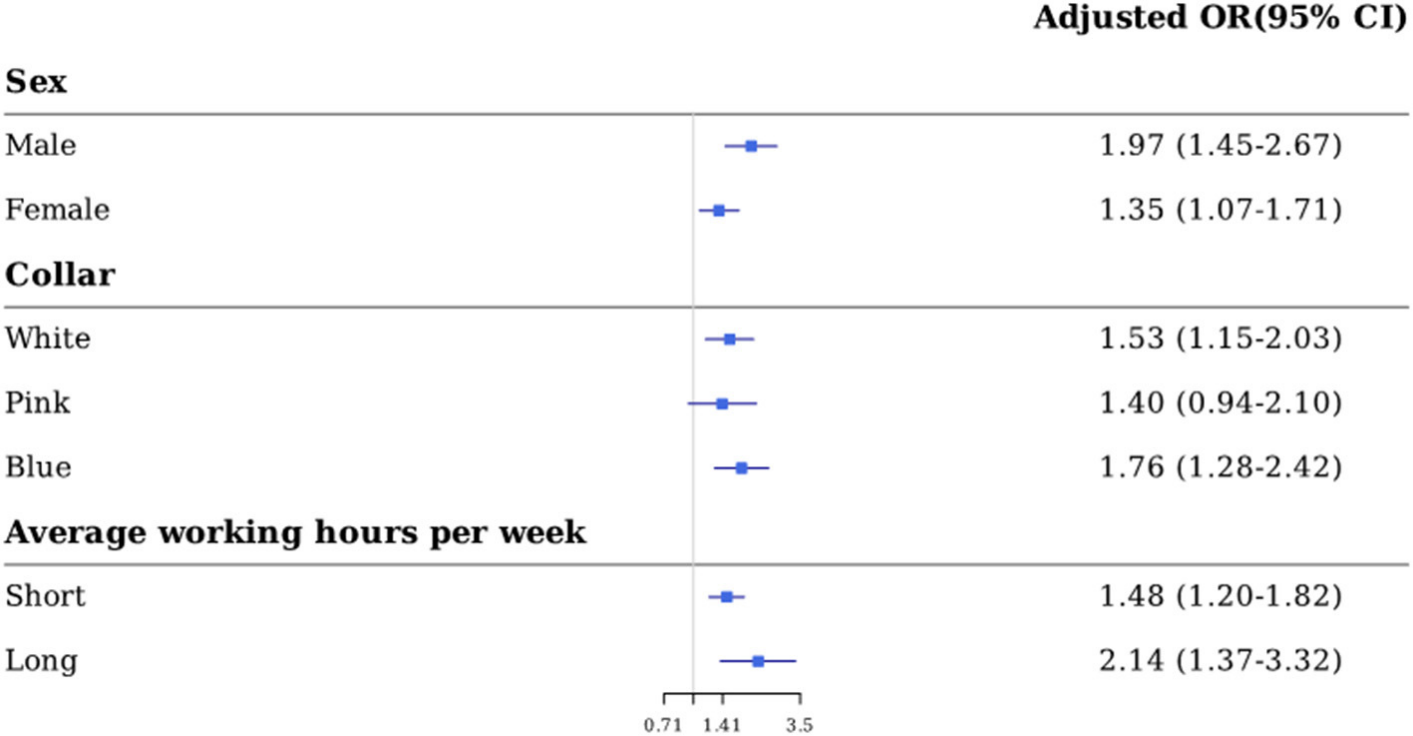
Stratified analysis for depression according to secondhand smoke exposure. Adjusted for age, sex, obesity, exercise, monthly drinking, severe drinking, income, education status, marriage status, weekly working hours, shift work, collar except for the stratification variable in each model.

In the stratification analysis results summarized in Figure 3, the completely adjusted ORs of depression by occupational SHS exposure were statistically significant for variables, including sex (male or female), occupation (white-collar and blue-collar), average working hours per week (long and short average working hours per week). In terms of sex, adjusted ORs (95% CIs) were 1.97 (1.45–2.67) for male and 1.35 (1.07–1.71) for female. ORs of white-collar workers and blue-collar workers were 1.53 (1.15–2.03) and 1.76 (1.28–2.42), respectively. Short average working hours group and long average working hours group had ORs of 1.48 (1.20–1.82) and 2.14 (1.37–3.32), respectively. Pink-collar workers showed borderline results, with the OR (95% CIs) of 1.40 (0.94–2.10).

## Discussion

This study found a significant correlation between occupational SHS exposure in the workplace and depression, and this association did not attenuate even after adjusting for various potential covariates. Additionally, we analyzed the association between exposure to SHS and depression by years. We found that the ORs showed a tendency to increase in the following years.

In 2014, 2016, and 2018, the policy was strengthened and the SHS exposure prevalence gradually decreased to 21.9, 10.6, and 8.71%, respectively. However, workers who never smoked are still exposed to smoke, so they feel that their right to work in clean air is violated because they are exposed to smoke involuntarily. SHS, unlike active smoking, renders an individual unable to self-selectively stop smoking, so it might have a greater impact on depression (31). We speculate that the gradual increase in depression observed in this study is influenced by the stress experienced by workers in having their right to clean air violated.

Furthermore, we summarized the prevalence of SHS by socioeconomic factors including education and income in Supplementary Figures 1, 2. In terms of education and income, the prevalence of SHS was in a decreasing trend from 2014 to 2018. The extent of decreasing prevalence was similar, regardless of income and education level. This may suggest that the increasing trend of OR of depression by SHS is not related to education or income. Thus, it is necessary to reduce the prevalence of SHS and reduce depression in the workplace through efficient policy.

Several studies have reported the association between SHS and depression. However, these results are inconsistent. A meta-analysis clarified the association between SHS and depression in the workplace (OR = 1.53) (32). A study (33) reported that SHS exposure had a significant relationship with depressive symptoms and stress. In contrast, the association between SHS and depression shows somewhat different results in (22), which is inconsistent with (33). These studies lacked valid outcomes as the data were generally obtained by a single or invalidated questionnaire. In our study we used the PHQ-9 to assess depression as the outcome variable.

Moreover, our study shows an interesting pattern of increased ORs of depression due to SHS, while the prevalence of SHS exposure was decreasing (Supplementary Table 1). Since several studies have shown that SHS negatively affects workers' health, the Korean government established law policies and guidelines with several amendments (19). This may result in decreased SHS exposure, however, this might also cause relative deprivation to workers who are exposed. The disparities in socioeconomic status and education level are significant in relation to those in SHS (34). The association of SHS with depression was still significant after adjusting for education and income, however, there might be unmeasured confounders generated by socioeconomic disparities. Therefore, additional policies must focus on the most vulnerable workers exposed to SHS.

A previous study (35) found that the greater the exposure time to SHS, the higher the ORs for depression. Also, the OR of women was higher than that of men. This is in agreement with our results, however, detailed results were not obtained because no further analysis was performed. To analyze in the occupational perspective, multivariate stratified logistic regression analysis was performed separately by year, sex, average working hours per week, and occupation. In stratification analysis, the association between SHS and depression was significant except for the borderline result in pink-collar workers. We found that the ORs of depression for SHS were high for those with prolonged work hours and for men. The difference in OR after gender stratification is not easily explained by reviewing the previous study (36). A discussion of this study highlighted the gender differences in the incidence of depression, finding that a lack of authority in the workplace was related to depression in men but not in women. We speculate that men feel deprived of their authority to control their environmental exposure to the harmful effects of passive smoking, making them more vulnerable to passive smoking related depressive symptoms, as our results showed.

We also found that passive smoking exposure was more closely related to depressive symptoms in the group with long working hours. Long working hours generally mean a longer duration of exposure, so our current results may suggest a dose response relationship between SHS and depressive symptoms. Therefore, men, especially those with prolonged working hours, who could be exposed to SHS for extended periods, are at high risk.

Analysis by occupational category was conducted, and significant results were obtained for both white- and blue-collar workers, however, pink-collar workers remained borderline. This may be due to the small number of pink-collar workers (*n* = 969) compared with white- and blue-collar workers (white-collar: 2,572, blue-collar: 1,675), and most of the pink-collar workers were women (81.5%).

Our study has several strengths. First, the relationship between passive smoking in the workplace and depression with occupational and work environmental factors was examined using stratified analysis. Occupational category and working hours, the most representative features in the working environment, were used as stratifying variables. Second, by using nationwide validated survey data, the study design is well-constructed with a large sample size. As participants in this survey were randomly selected from the general population, the results can be generalized to the entire population. Third, we adjusted for many variables, including demographic factors (age and sex), work environmental factors (occupation category, working hours per week, and shift work), and lifestyle factors (BMI, smoking, alcohol consumption, and physical activity).

Nevertheless, there are some limitations interpreting the results of our study. First, this is a cross-sectional study and could not reflect any causal relationship between passive smoking and depression. However, since passive smoking happens unintentionally and people who never smoked were excluded from this study, we could infer a temporal relationship between passive smoking and depression. Further studies that elucidate the causal association of passive smoking with depression should be implemented. Second, because the survey was conducted via interviews with the participants using self-reported data, recall and response bias may have occurred. This is an inevitable limitation, however, this may be interpreted as a random error, compensated by a large sample size. Third, the data were restricted to South Korea and could not be generalized for race or ethnicity. Further multinational studies could overcome this limitation. Fourth, a bias could have occurred due to a lack of information with respect to the workplace environment, including its own smoking policy, physical/mental abuse, and the existence of a protocol.

In conclusion, this study clarifies the association of passive smoking in the workplace with the prevalence of depression. Even though workers' exposure to passive smoking in the workplace decreases with improved policies, there is a need for constant attention to the mental health of vulnerable workers exposed to passive smoking in the workplace.

## Data Availability Statement

Publicly available datasets were analyzed in this study. This data can be found here: https://knhanes.kdca.go.kr/knhanes/sub03/sub03_02_05.do.

## Ethics Statement

The studies involving human participants were reviewed and approved by Severance Hospital. Written informed consent to participate in this study was provided by the participants' legal guardian/next of kin.

## Author Contributions

SK and JO: conceptualization and formal analysis. BY and J-HY: methodology and project administration. JO and AC: software. J-HY and JS: validation and writing—review and editing. J-HY: investigation, resources, and funding acquisition. SK, BY, and JS: data curation. BY, SK, and JO: writing—original draft preparation. SK, AC, and JS: visualization. JO and J-HY: supervision. All authors contributed to the article and approved the submitted version.

## Funding

This work was supported by the Korea Health Industry Development Institute through “Korea Health Promotion Research and Development” funded by the Ministry of Health & Welfare (HS21C2367).

## Conflict of Interest

The authors declare that the research was conducted in the absence of any commercial or financial relationships that could be construed as a potential conflict of interest.

## Publisher's Note

All claims expressed in this article are solely those of the authors and do not necessarily represent those of their affiliated organizations, or those of the publisher, the editors and the reviewers. Any product that may be evaluated in this article, or claim that may be made by its manufacturer, is not guaranteed or endorsed by the publisher.

## Abbreviations

SHS: Secondhand smoke
OR: Odds ratio
CI: Confidence interval.
