# Supplementary figures and images for: Secondhand Smoke in the Workplace Is Associated With Depression in Korean Workers
Source: Front Public Health. 2022 Apr 26;10:802083. doi: 10.3389/fpubh.2022.802083 (PMC9087188; doi:10.3389/fpubh.2022.802083)

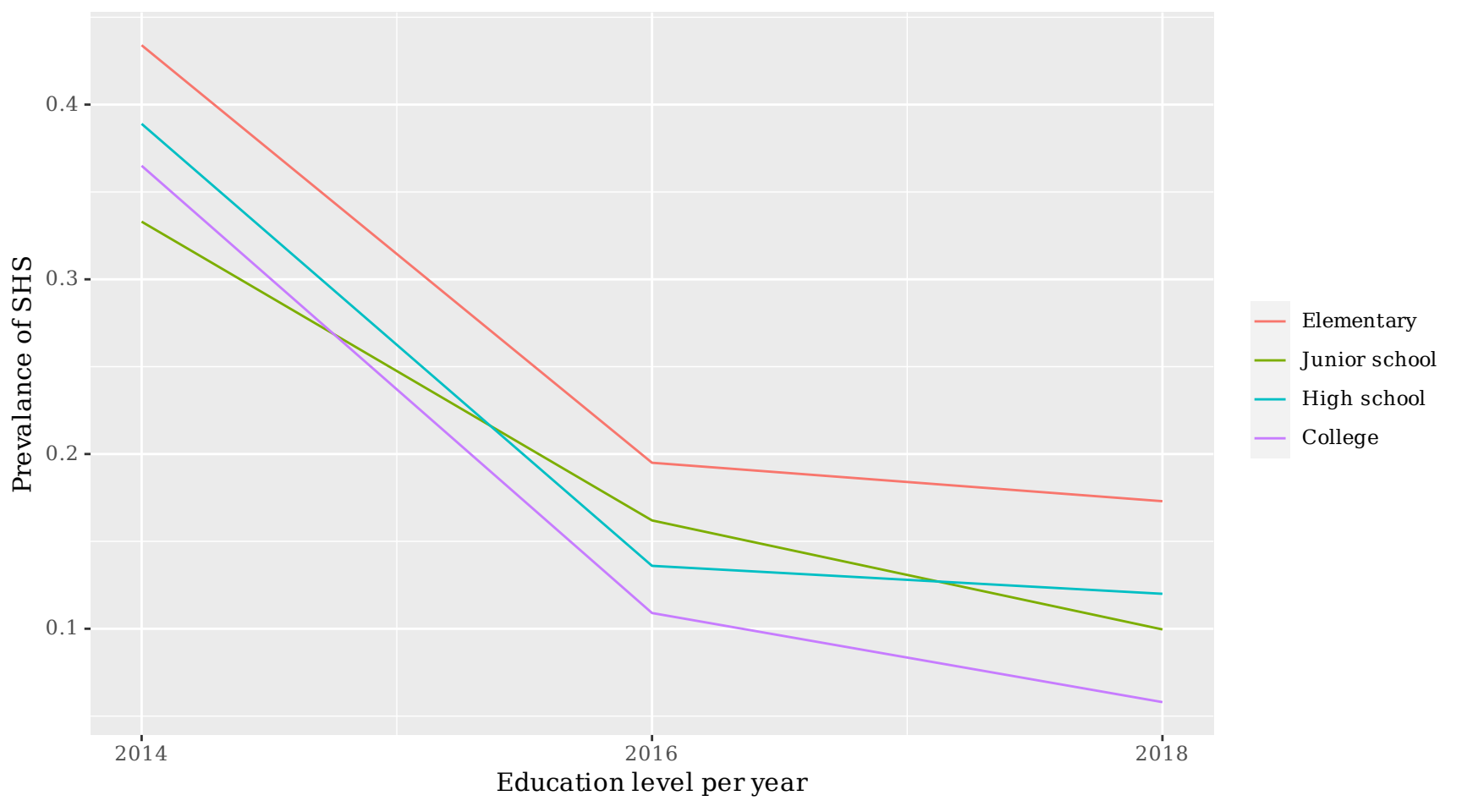

Supplement: Supplementary file 2 [file Image_1.PDF]

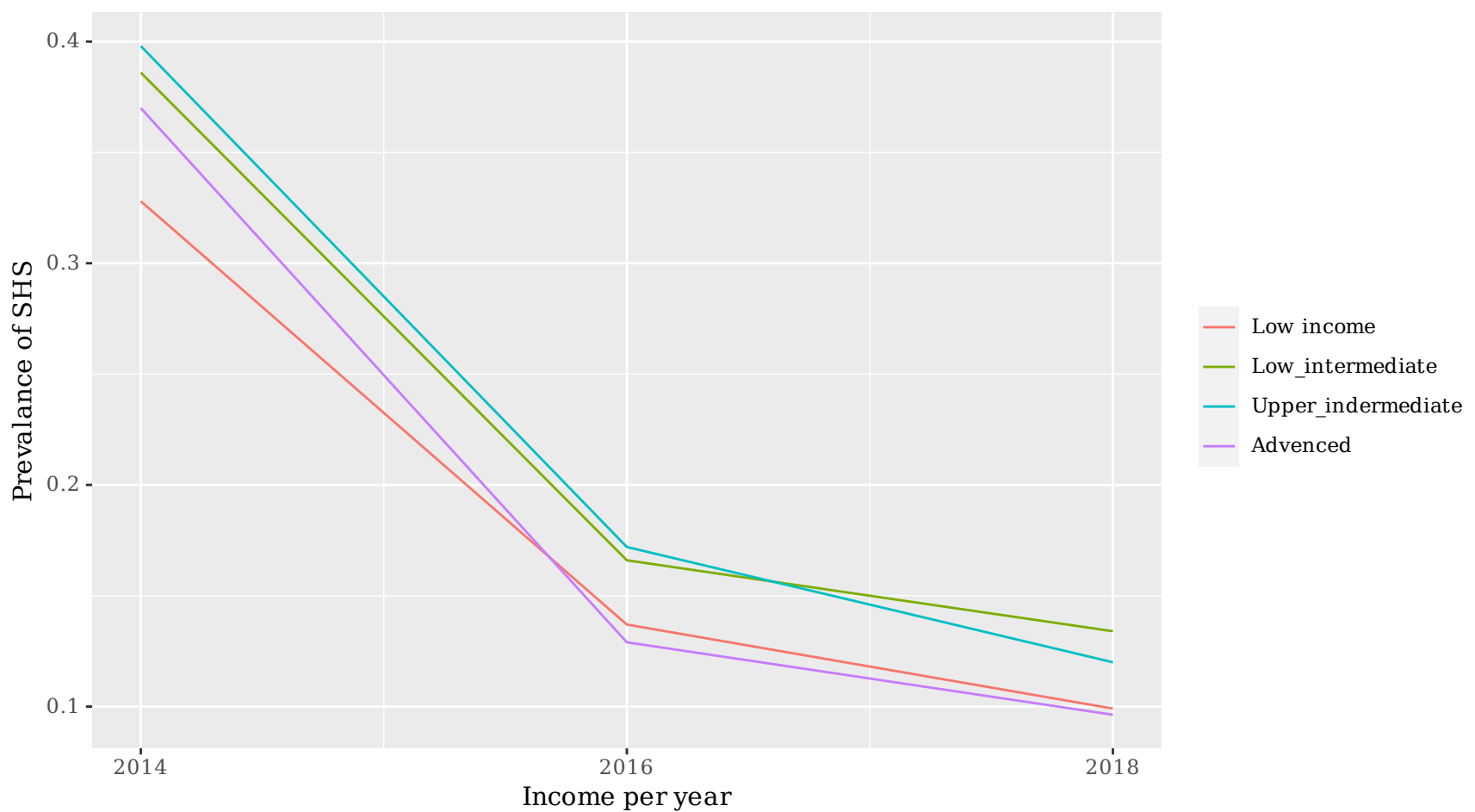

Supplement: Supplementary file 3 [file Image_2.PDF]
